# Supplementary material for: Trichosanthin Promotes Anti-Tumor Immunity through Mediating Chemokines and Granzyme B Secretion in Hepatocellular Carcinoma
Source: Int J Mol Sci. 2023 Jan 11;24(2):1416. doi: 10.3390/ijms24021416 (PMC9864620; doi:10.3390/ijms24021416)
Supplement: Supplementary file 1 [file ijms-24-01416-s001.zip › ijms-2102185-supplementary.pdf]

Supplementary Materials:

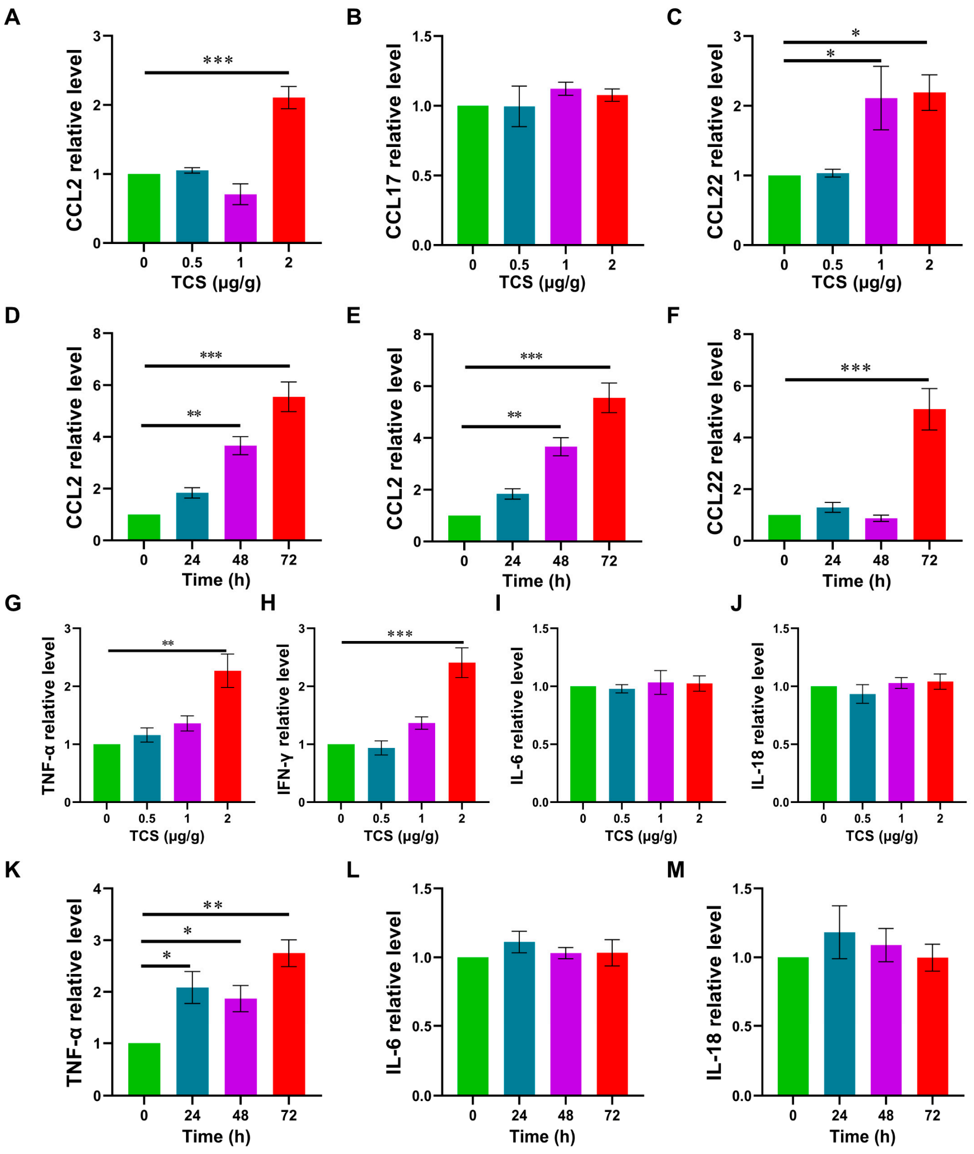

Figure S1. mRNA expression of CCL2, CCL17, CCL22, TNF-α, IFN-γ, IL-6, and IL-18. (**A-C**) RT-qPCR method detected mRNA levels of CCL2, CCL17 and CCL22 in HCC tissues treated with different doses of TCS (0, 0.5, 1 and 2 μg/g), GAPDH as the internal reference gene; (**D-F**) RT-qPCR method detected the mRNA levels of CCL2, CCL17 and CCL22 in H22 HCC cells treated with 25 μg/g TCS after 24h, 48h and 72h, GAPDH as the internal reference gene; (**G-J**) RT-qPCR method detected the mRNA levels of TNF-α, IFN-γ, IL-6, and IL-18 in HCC tissues treated with different doses of TCS (0, 0.5, 1 and 2 μg/g), GAPDH as the internal reference gene; (**K-M**) RT-qPCR method detected the mRNA levels of TNF-α, IL-6, and IL-18 in H22 HCC cells treated with 25 μg/g TCS after 24h, 48h and 72h, GAPDH as the internal reference gene. *, $p < 0.05$; **, $p < 0.01$; ***, $p < 0.001$.

Table S1. The antibody information in this study.

	Clones	Manufacturer	Host / Isotype	Conjugation
Caspase 3	Polyclonal antibody	Cell Signaling, cat# 9662	Rabbit / IgG	Unconjugated
Caspase 8	Polyclonal antibody	Abclonal, A0215	Rabbit / IgG	Unconjugated
Caspase 9	Polyclonal antibody	Abclonal, A7523	Rabbit / IgG	Unconjugated
Cleaved-caspase 3	Monoclonal antibody (5A1E)	Cell Signaling, cat# 9664T	Rabbit / IgG	Unconjugated
Cleaved-caspase 8	Monoclonal antibody (D5B2)	Cell Signaling, cat# 8592T	Rabbit / IgG	Unconjugated
Cleaved-caspase 9	Polyclonal antibody	Cell Signaling, cat# 9509T	Rabbit / IgG	Unconjugated
Granzyme B	Monoclonal antibody (D2H2F)	Cell Signaling, cat# 17215S	Rabbit / IgG	Unconjugated
M6PR	Monoclonal antibody (D3V8C)	Cell Signaling, cat# 14364S	Rabbit / IgG	Unconjugated
LC3B	Polyclonal antibody	Cell Signaling, cat# 2775S	Rabbit / IgG	Unconjugated
P62	Polyclonal antibody	Proteintech, 18420-1-AP	Rabbit / IgG	Unconjugated
GAPDH	Monoclonal antibody (1E6D9)	Proteintech, 60004-1-Ig	Mouse/IgG2b	Unconjugated
PARP	Monoclonal antibody (46D11)	Cell Signaling, cat# 9532	Rabbit / IgG	Unconjugated
Cleaved PARP	Polyclonal antibody	Cell Signaling, cat# 9544	Rabbit / IgG	Unconjugated
Ki-67	Monoclonal antibody (D3B5)	Cell Signaling, cat# 9129	Rabbit / IgG	
CoraLite® Plus 488-conjugated CD8 Monoclonal antibody	Monoclonal antibody (1G2B10)	Proteintech, CL488-66868	Mouse / IgG1	CoraLite® Plus 488 Fluorescent Dye

Donkey anti-Rabbit IgG (H+L) Highly Cross-Adsorbed Secondary Antibody	Polyclonal	ThermoFisher Scientific	Donkey / IgG	Alexa Fluor ™ 555

Table S2. Sequences of primers for real-time PCR.

Primer		Sequence (5′ to 3′)
CCL2	CCL2-qPCR-F	TTAAAAACCTGGATCGGAACCAA
	CCL2-qPCR-R	GCATTAGCTTCAGATTTACGGGT
CCL22	CCL22-qPCR-F	AGGTCCCTATGGTGCCAATGT
	CCL22-qPCR-R	CGGCAGGATTTTGAGGTCCA
CCL17	CCL17-qPCR-F	TACCATGAGGTCACTTCAGATGC
	CCL17-qPCR-R	GCACTCTCGGCCTACATTGG
IFN-γ	IFNγ-qPCR-F	ATGAACGCTACACACTGCATC
	IFNγ-qPCR-R	CCATCCTTTTGCCAGTTCCTC
IL18	IL18-qPCR-F	GACTCTTGCGTCAACTTCAAGG
	IL18-qPCR-R	CAGGCTGTCTTTTGTCAACGA
IL-6	IL6-qPCR-F	TAGTCCTTCCTACCCCAATTTCC
	IL6-qPCR-R	TTGGTCCTTAGCCACTCCTTC
TNF-α	TNFα-qPCR-F	CCTGTAGCCCACGTCGTAG
	TNFα-qPCR-R	GGGAGTAGACAAGGTACAACCC
GAPDH	GAPDH-qPCR-F	AGGTCGGTGTGAACGGATTTG
	GAPDH-qPCR-R	TGTAGACCATGTAGTTGAGGTCA
